# Intensive expression of Bmi-1 is a new independent predictor of poor outcome in patients with ovarian carcinoma

**DOI:** 10.1186/1471-2407-10-133

**Published:** 2010-04-08

**Authors:** Guo-Fen Yang, Wei-Peng He, Mu-Yan Cai, Li-Ru He, Jun-Hang Luo, Hai-Xia Deng, Xin-Yuan Guan, Mu-Sheng Zeng, Yi-Xin Zeng, Dan Xie

**Affiliations:** 1 State Key Laboratory of Oncology in South China, Cancer Center, Sun Yat-Sen University, No. 651, Dongfeng Road East, 510060 Guangzhou, China; 2 Department of Gynecology, the First Affiliated Hospital, Sun Yat-Sen University, No. 78, Zhongshan Road II, 510080 Guangzhou, China

## Abstract

**Background:**

It has been suggested that the B-cell specific moloney leukemia virus insertion site 1 (Bmi-1) gene plays an oncogenic role in several types of human cancer, but the status of *Bmi-1 *amplification and expression in ovarian cancer and its clinical/prognostic significance are unclear.

**Methods:**

The methods of immunohistochemistry and fluorescence *in situ *hybridization were utilized to examine protein expression and amplification of *Bmi-1 *in 30 normal ovaries, 30 ovarian cystadenomas, 40 borderline ovarian tumors and 179 ovarian carcinomas.

**Results:**

Intensive expression of Bmi-1 was detected in none of the normal ovaries, 3% cystadenomas, 10% borderline tumors, and 37% ovarian carcinomas, respectively. Amplification of *Bmi-1 *was detected in 8% of ovarian carcinomas. In ovarian carcinomas, significant positive associations were found between intensive expression of Bmi-1 and the tumors ascending histological grade, later pT/pN/pM and FIGO stages (*P *< 0.05). In univariate survival analysis of the ovarian carcinoma cohorts, a significant association of intensive expression of Bmi-1 with shortened patient survival (mean 49.3 months *versus *100.3 months, *p *< 0.001) was demonstrated. Importantly, Bmi-1 expression provided significant independent prognostic parameters in multivariate analysis (*p *= 0.005).

**Conclusions:**

These findings provide evidence that intensive expression of Bmi-1 might be important in the acquisition of an invasive and/or aggressive phenotype of ovarian carcinoma, and serve as a independent biomarker for shortened survival time of patients.

## Background

Ovarian cancer is a major lethal gynecological malignancy worldwide [1]. Its peak incidence is at the age 45 or above. Because of its insidious onset, approximately 70% of ovarian cancer patients were diagnosed at advanced stage(FIGO III/IV stage) with a very poor prognosis, whose 5-year survival rate is of <30% [2]. Ovarian carcinoma is the most common histopathological type of ovarian cancer. The development and progression of ovarian carcinoma are presumed to be a multi-step process involving multiple genetic changes [3]. Thus, a substantial amount of research on ovarian carcinoma has focused on the discovery of specific molecular markers that are present in ovarian carcinoma cells which could serve as reliable prognostic factors.

The B-cell specific moloney leukemia virus insertion site 1 (Bmi-1) gene belongs to mammalian Polycomb-group (PcG) family forming multimeric gene-repressing complexes involved in axial patterning, hematopoiesis, regulation of proliferation, and senescence. *Bmi-1 *was first identified as a proto-oncogene that cooperated with c-Myc in generating pre-B-cell lymphomas in a murine model [4-8]. It has been discovered that Bmi-1 participates in cell cycle regulation by acting as a stable transcriptional repressor of the Ink4a locus, which encodes the tumor suppressor proteins p16Ink4a and p19Arf (mouse homologue of human p14ARF). Inactivation of the p16Ink4a-pRb pathway and p14ARF-MDM2-p53 pathway by Bmi-1 deregulation has been clearly implicated in lymphomagenesis [9,10] and oncogenesis in nonsmall-cell lung cancer of human [11]. This suggested that the *Bmi-1 *gene plays an important role in cell proliferation and tumor progression. It has been confirmed that *Bmi-1 *gene is widely expressed in diverse human tumors, including non-small cell lung cancer, hepatocellular carcinoma, B-cell non-Hodgkin's lymphoma, breast cancer, ovarian cancer, colorectal cancer, skin cancer and neuroblastoma [10-20], and has been shown to be a useful prognostic marker in myelodysplastic syndrome and many cancers, including nasopharyngeal carcinoma, bladder cancer and gastric cancer [17-20].

To date, however, the status of Bmi-1 expression and its clinical/prognostic relevance in ovarian cancer have not been fully elucidated. In this study, the protein expression and amplification status of *Bmi-1 *in a series of human epithelial ovarian tissue, normal and pathological, non-neoplastic and neoplastic, were examined. The clinico-pathological and prognostic significance of expression of Bmi-1 in our ovarian carcinoma cohorts was also assessed.

## Methods

### Patients and tissue specimens

In this study, a total of 249 epithelial ovarian tumors (benign, borderline and carcinomatous) were obtained from archives of paraffin-embedded tissues between 1996 and 2008 at the Department of Pathology, Cancer Center and the First Affiliated Hospital, Sun Yat-Sen University, Guangzhou, China. The cancer cases selected were based on availability of resection tissue and follow-up data. Patients whose cause of death remained unknown were excluded from our study. The ovarian tumor cases encompassed 179 histologically confirmed invasive carcinomas, 40 borderline tumors and 30 cystadenomas. Data of survival time and clinico-pathological parameters were collected. Ages of the 179 patients with ovarian carcinoma ranged from 18 to 86 years (mean age, 50.7 years) and their clinico-pathological characteristics are summarized in Table 1. None of the cancer patients in this study had received preoperative radiation or chemotherapy. In addition, 30 specimens of normal ovaries from exairesis for non-ovary diseases in the Department of Gynaecology and Obstetrics of the First Affiliated Hospital, Sun Yat-Sen University from 2005 to 2008 were used as control. For the use of these clinical materials for research purposes, prior patient's consent and approval from the Institute Research Medical Ethics Committee of Sun Yat-Sen University was obtained.

**Table 1:** Association of Bmi-1 expression with patient's clinico-pathological features in ovarian carcinomas

		Bmi-1 protein		
		
	All cases	Low expression	Intensive expression	*P *value^a^
Age at surgery (years)				0.452
≤ 50.7^b^	92	60 (65%)	32 (35%)	
> 50.7	87	52 (60%)	35 (40%)	
Histological type				0.038
Serous (grade 1)	11	9 (82%)	2 (18%)	
Serous (grade2/3)	107	68 (64%)	39 (36%)	
Mucinous	23	15 (65%)	8 (35%)	
Endometrioid	8	7 (88%)	1 (12%)	
Clear cell	7	5 (71%)	2 (29%)	
Undifferentiated	23	8 (35%)	15 (65%)	
Histological grade (Silveberg)				0.011
G1	36	29 (81%)	7 (19%)	
G2	101	63 (62%)	38 (38%)	
G3	42	20 (48%)	22 (52%)	
pT status				0.037
pT1	51	39 (76%)	12 (24%)	
pT2	35	22 (63%)	13 (37%)	
pT3	93	51 (55%)	42 (45%)	
pN status				0.001
pN0	88	66 (75%)	22 (25%)	
pN1	91	46 (51%)	45 (49%)	
pM status				0.006
pMX	153	102 (67%)	51 (33%)	
pM1	26	10 (38%)	16 (62%)	
FIGO stage				0.002
I	33	27 (82%)	6 (18%)	
II	21	17 (81%)	4 (19%)	
III	99	58 (59%)	41 (41%)	
IV	26	10 38%)	16 (62%)	

^a^Chi-square test

^b^Mean age

### Construction of tissue microarrays (TMA)

The TMA was constructed according to a method described previously [21]. Briefly, the individual donor tissue block and the corresponding histological H&E stained slides were overlaid for tissue TMA sampling. The tissues (179 ovarian carcinoma, 40 borderline tumor, and 30 cystadenoma tissues) were sampled using a tissue arraying instrument (Beecher Instruments, Silver Spring, MD); a 0.6-mm-diameter cylinder of tissue was removed. Subsequently, the tissue cylinder was re-embedded into a predetermined position in a recipient paraffin block. In our constructed ovarian tumor tissue-TMA, 3 cores of sample were selected from each tumor tissue. Multiple sections (5 μm thick) were cut from the TMA block and mounted on microscope slides.

### Immunohistochemistry (IHC)

IHC studies were performed using a standard streptavidin-biotin-peroxidase complex method [22]. For antigen retrieval, tissue slides were microwave-treated and boiled in a 10 mM citrate buffer (pH 6.0) for 10 min. The slides were incubated with rabbit monoclonal antibody against human Bmi-1 (Shangying Biotechnology Inc. Wuhan, China, 1:2000 dilution), mouse monoclonal antibodies against human p16Ink4a (1:200 dilution) or p14ARF (1:300 dilution, Labvision Co., Neomarkers, USA) overnight at 4°C in a moist chamber. A negative control was obtained by replacing the primary antibody with normal rabbit or mouse IgG. Known immunostaining positive slides were used as positive controls.

**Figure 1:**
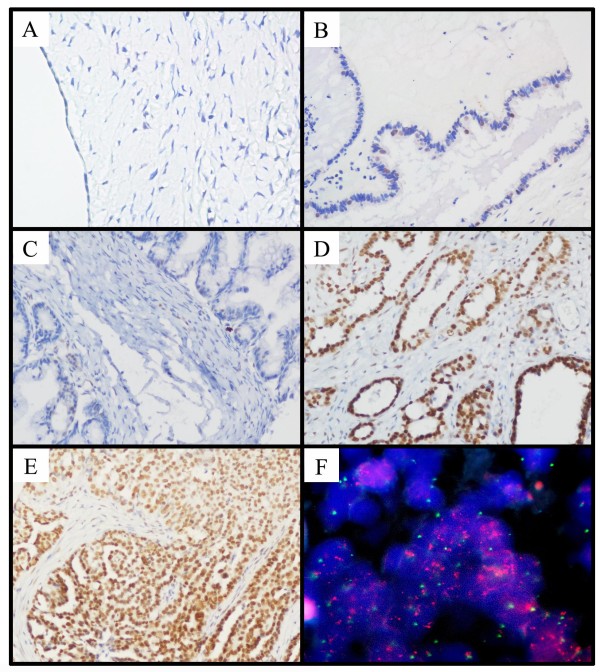
**Immunohistochemical staining of Bmi-1 protein and Fluorescence *in situ *hybridization of *Bmi-1 *gene in human ovarian tissues**. (A) Negative expression of Bmi-1 was observed in a normal surface epithelium of ovary (200×). (B) An ovarian cystadenoma showed low expression of Bmi-1, in which about 10% of tumor cells was detected moderate positive staining of Bmi-1 (200×). (C) Low expression of Bmi-1 was observed in an borderline ovarian tumors, in which 5% of tumor cells showed weak positive staining of Bmi-1 (200×). (D) Intensive expression of Bmi-1 was detected in an ovarian carcinoma (case 33), in which more than 90% of carcinoma cells showed strong positive staining of Bmi-1 (200×). (E) Another ovarian carcinoma (case 161) showed intensive expression of Bmi-1, in which all of tumor cells had strong positive staining of Bmi-1 (200×). (F) Amplification of *Bmi-1 *gene was observed by FISH in the same ovarian carcinoma case (161), in which *Bmi-1 *gene signals (red) was detected at least 3 times more than centromere signals of chromosome 10 (green) (1000×).

Expression levels of Bmi-1 protein were visualized by observing the stained tissues under a light microscope. Positive expression of Bmi-1 was defined as the presence of brown or yellowish brown granules in the nuclei, though occasionally yellowish brown granules could also be seen in the cytoplasm. For evaluation of the Bmi-1 IHC staining in different ovarian tissues, a semi-quantitative scoring criterion for IHC of Bmi-1 described previously [16] was used, in which both staining intensity and positive areas were recorded. A staining index (values 1 to 16), obtained as the intensity of Bmi-1 positive staining (negative = 1, weak = 2, moderate = 3, or strong = 4 scores) and the proportion of immunopositive cells of interest (≤ 10% = 1, >10% to ≤ 50% = 2, >50% to ≤ 75% = 3, >75% = 4 scores) were calculated. The two different scores of corresponding sample were then multiplied. Points equal or less than 4 was marked as (-); 4 points to 8 points was marked as (+); 8 points to 12 points, (++); and 12 points to 16 points, (+++). For statistical analysis, (-) and (+) were counted as low expression of Bmi-1 (Fig. 1A, B and 1C), while (++) and (+++) were counted as intensive expression of Bmi-1 (Fig. 1D and 1E). The evaluation of p16Ink4a and p14ARF IHC staining was scored on a semi-quantitative scale [14], as follows: negative expression (<10% of the cells were positive), down-regulated expression (small cell clusters, but 10-50% of the cells were positive) and diffuse expression (>50% of the cells were positive). Results were observed and assessed by two independent pathologic doctors, without knowing the identity of the samples.

### Fluorescence in situ hybridization (FISH)

Two-color FISH was performed using a Spectrum Orange-labeled BAC clone (RP11-573G6) at 10p12 containing the *Bmi-1 *gene and a Spectrum Green-labeled reference centromeric probe on chromosome 10 (Vysis, Downers Grove, IL). The FISH reaction was performed as described previously [23] with slight modification. Briefly, deparaffinized ovarian carcinoma tissue sections were treated with proteinase K (400 μg/ml) at 37°C for 30 min, followed by denaturing in 70% formamide, 2× SSC at 75°C for 6 min. Fifty nanograms of each probe were mixed in a 20 μl hybridization mixture (containing 55% formamide, 2× SSC, and 2 ∝g human Cot1 DNA), denatured at 75°C for 6 min and then hybridized to the denatured tissue sections at 37°C for 24 hours. The slides were counterstained with 1 μg/ml DAPI in an anti-fade solution and were examined with a Zeiss Axiophot microscope equipped with a triple-band pass filter. A minimum of 300 tumor cells was evaluated per specimen. Amplification of *Bmi-1 *was defined as presence of either 6 (or more) *Bmi-1 *gene signals or at least 3 times as many gene signals than centromere signals of chromosome 10 in tumor cells (Fig. 1F).

### Statistical analyses

Statistical analysis was carried out with the SPSS statistical software package (SPSS Standard version 13.0, SPSS Inc.). The association of Bmi-1 protein expression with ovarian carcinoma patient's clinico-pathological features was assessed by the Chi-square test. Multivariate survival analysis was performed on all parameters that were found to be significant on univariate analysis using the Cox regression model. For univariate survival analysis, we analyzed all ovarian carcinoma patients by Kaplan-Meier analysis. Log rank test was used to compare different survival curves. *P *< 0.05 was considered significant.

## Results

### Bmi-1 expression in ovarian tissues

Bmi-1 expression could be evaluated informatively in TMA tissues of 163/179 of ovarian carcinomas, 37/40 of borderline tumors and 26/30 of cystadenomas. The non-informative TMA samples included unrepresentative samples, samples with too few tumor cells (<300 cells per case) and lost samples. IHC staining of such non-informative samples were replaced and performed by using whole tissue slides. In our study, we defined that (-) and (+) were counted as low expression of Bmi-1, while (++) and (+++) were counted as intensive expression of Bmi-1. According to this definition, the intensive expression of Bmi-1 was detected in 67/179 (37%) ovarian carcinomas. The increasing frequencies of Bmi-1 intensive expression in normal ovarian epithelium (0), to benign cystadenomas (3%), to borderline tumors (10%), and to invasive carcinomas were significant (37%, *P *< 0.05, Table 2).

**Table 2:** The expression of Bmi-1 in normal ovaries and in benign and malignant epithelial ovarian tumors^a^

		Bmi-1 protein	
	
	All cases	Low expression	Intensive expression
Normal ovaries	30	30 (100%)	0 (0)
Cystadenomas	30	29 (97%)	1 (3%)
Borderline tumors	40	36 (90%)	4 (10%)
Invasive carcinomas	179	112 (63%)	67 (37%)

^a^Values are n (%). A significant increasing frequency of intensive expression of Bmi-1 was observed in cystadenomas, in borderline tumors and in invasive carcinomas (*P *< 0.01, Chi-Square Test for Trend)

### Association of Bmi-1 expression with ovarian carcinoma patient clinico-pathologic features

The association between Bmi-1 expression in ovarian carcinomas and several known clinico-pathological features was further studied. Bmi-1 expression was positively correlated with tumors histological type, grade, pT/pN/pM status, and FIGO stage (*P *< 0.05, Table 2). No significant correlation was obtained between Bmi-1 expression and patient age (≤ 50.7 years *vs *> 50.7 years) (*P *> 0.05, Table 1).

### Relationship between clinicopathologic variables, Bmi-1 expression and ovarian carcinoma patient survival: Univariate survival analysis

**Figure 2:**
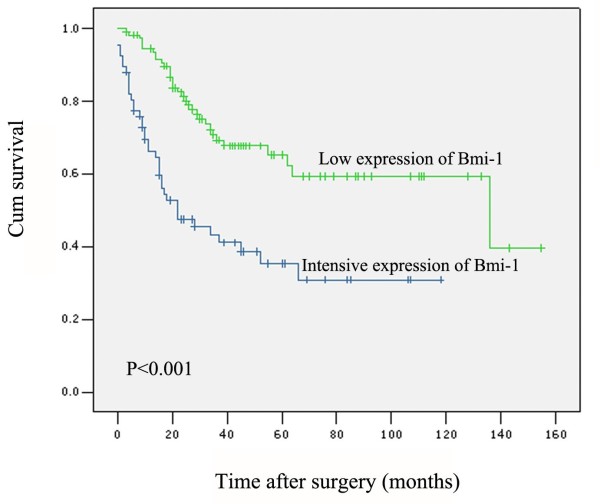
**Kaplan-Meier survival analysis according to Bmi-1 expression in 179 patients with invasive ovarian carcinoma (log-rank test)**. Probability of survival of patients: low expression of Bmi-1, *n *= 112; intensive expression of Bmi-1, *n *= 67 (*p *< 0.001).

In univariate survival analyses, cumulative survival curves were calculated according to the Kaplan-Meier method. Differences in survival times were assessed with the log-rank test. First, to confirm the representativeness of the ovarian carcinomas in our study, we analyzed established prognostic predictors of patient survival. Kaplan-Meier analysis demonstrated a significant impact of well-known clinical pathological prognostic parameters, such as tumor histological grade (*p *= 0.012), pT/pN/pM status (*p *< 0.01) and FIGO stage (*p *< 0.001) on patient survival (Table 3). The mean survival time for patients with tumors having intensive expression of Bmi-1 was 49.3 months compared to 100.3 months for pateints with tumors having low expression of Bmi-1 (*P *< 0.001, Fig. 2, Table 3). In stratified survival analysis, Bmi-1 expression could stratify the outcome of patients in hige-grade (grade 2/3) serous carcinoma (*P *= 0.045), mucinous carcinoma (*P *= 0.001) and undifferentiated carcinoma (*P *= 0.001) subgroups.

**Table 3:** Clinical pathological parameters and expression of Bmi-1 for prognosis of 179 patients with ovarian carcinoma by univariate survival analysis (log-rank test)

Variable	All cases	Mean survival (months)	Median survival (months)	*P *value
Age at surgery (years)				0.388
≤ 50.7^a^	92	83.4	136.0	
> 50.7	87	81.7	55.0	
Histological type				0.491
Serous(grade 1)	11	110.4	136.0	
Serous(grade2/3)	107	63.4	52.0	
Mucinous	23	75.8	NR^b^	
Endometrioid	8	116.7	NR	
Clear cell	7	102.8	NR	
Undifferentiated	23	32.7	NR	
Histological grade (Silveberg)				0.012
G1	36	104.9	136.0	
G2	101	85.9	64.0	
G3	42	48.0	29.0	
pT status				0.003
pT1	51	109.5	NR	
pT2	35	82.2	NR	
pT3	93	66.5	35.0	
pN status				<0.001
pN0	88	100.9	136.0	
pN1	91	52.8	28.0	
pM status				<0.001
pMX	153	94.8	136.0	
pM1	26	21.5	9.0	
FIGO stage				<0.001
I	33	134.2	NR	
II	21	115.0	NR	
III	99	71.5	37.0	
IV	26	21.5	9.0	
Bmi-1 expression				<0.001
Low	112	100.3	136.0	
Intensive	67	49.3	22.0	

^a^Mean age

^b^Not reached

### Independent prognostic factors of ovarian carcinoma: Multivariate Cox regression analysis

Since variables observed to have prognostic influence by univariate analysis may covariate, the expression of Bmi-1 as well as other clinical pathological parameters that were significant in univariate analysis (pN stage and FIGO stage) was examined in multivariate analysis (Table 4). The expression of Bmi-1 was found to be an independent prognostic factor for poor overall survival (relative risk: 1.998, CI: 1.228-3.251, *P *= 0.005). Of the other parameters, pN stage (*P *= 0.010) and FIGO stage (*P *= 0.004) were also demonstrated as independent prognostic factor for overall survival.

**Table 4:** Multivariate analysis on overall survival (Cox regression model)

Variable	Relative risk	95% Confidence interval	*P *value
Bmi-1^a^	1.998	1.228-3.251	0.005
Histological grade^b^	0.940	0.614-1.439	0.777
pT status^c^	1.189	0.788-1.793	0.409
pN status^d^	2.016	1.184-3.432	0.010
pM status^e^	0.975	0.280-3.393	0.975
FIGO stage^f^	3.686	1.521-8.931	0.004

^a^Intensive expressin *vs *Low expression

^b^G1 *vs *G2 *vs *G3

^c^pT1 *vs *pT2 *vs *pT3

^d^pN0 *vs *pN1

^e^pMX *vs *pM1

^f^Stage I *vs *Stage II *vs *Stage III *vs *StageIV

### Amplification of Bmi-1 in ovarian tumor TMA

In our FISH study, the FISH analysis was informative in 96/179 of ovarian carcinomas, 21/40 of borderline ovarian tumors and 15/30 of ovarian cystadenomas. Samples without FISH signal and samples with weak target signals or those with a strong signal background were the main reasons for most of the non-informative cases. FISH results demonstrated that the amplification of *Bmi-1 *was not detected in any of the ovarian cystadenoma and borderline tumor tissues; but was detected in 8% (8/96) of the informative ovarian carcinomas; in each of the 8 cases with *Bmi-1 *amplification, intensive expression of Bmi-1 was observed (Fig. 1E and 1F). In the remaining 88 informative cancers without amplification of *Bmi-1*, 60 (68%) cases showed low expression of Bmi-1, while 28 (32%) cases were observed intensive expression of Bmi-1.

### Correlation between the expression of Bmi-1 and p16Ink4a and p14ARF in ovarian carcinomas

Since it was suggested that modulation of Bmi-1 protein might be involved in human colorectal carcinogenesis by repressing p16Ink4a/p14ARF proteins {14}, we further examined the expression of p16Ink4a/p14ARF by IHC in our ovarian carcinoma cohorts. By utilizing the criterion of a semi-quantitative scale as previously described [14], we found that 36% (64/179) and 17% (30/179) of ovarian carcinomas had negative/down-regulated expression of p16Ink4a and p14ARF, respectively. Further correlation analysis demonstrated that no significant correlation between Bmi-1 expression and expression of either p16Ink4a or p14ARF was evaluated in these ovarian carcinoma cohorts (*P *> 0.05, Fishers exact test).

## Discussion

In recent years, the incidence of ovarian carcinoma has been increasing in Asian countries such as China and Singapore [24]. The available clinicopathologic prognostic indicators are not accurate, although the treatment of ovarian cancer has been improved greatly in recent years, its 5-year survival rate is of <30% [2]. Thus, it is important to identify a biological genetic molecular marker that is associated with pathophysiologic processes of human ovarian cancer.

The gene, *Bmi-1*, was initially shown to regulate haematopoiesis and differentiation of lymphocytes [25] and to be involved in cerebral development [26]. To date, the proto-oncogene *Bmi-1 *has been reported to be up-regulated in a large number of neoplasias, namely in lymphomas [27], cerebral tumours [26], breast cancer [13] and other epithelial tumours [28,29] and to be an oncogene associated with poor prognosis in various tumours [12]. To investigate whether or not the abnormal expression of Bmi-1 is involved in the pathogenesis of ovarian carcinoma, in the present study, the protein expression of Bmi-1 was examined firstly by IHC in normal ovaries, benign and borderline epithelial ovarian tumors, and malignant epithelial cancers. The results demonstrated that the expression of Bmi-1 in all of the normal ovary specimens was absent or at low levels. In our ovarian tumor specimens, a significant increasing expression of Bmi-1 was observed from benign cystadenoma to borderline tumor, and to carcinoma. In addition, we found that the frequency of intensive expression of Bmi-1 in undifferentiated ovarian carcinomas was significantly larger than that in other types of carcinoma. In serous carcinomas, intensive expression of Bmi-1 was more likely to be observed in grade 2/3 tumors than that in grade 1 tumors. Furthermore, intensive expression of Bmi-1 in our ovarian carcinoma cohorts was strongly correlated with an ascending histological grade and clinical stage (pT/pN/pM and FIGO stage) of the tumor. These findings suggest that up-regulated expression of *Bmi-1 *in ovarian carcinoma may represent an acquired malignant phenotypic feature of tumor cells.

Datas from several clinical studies show that abnormal expression of *Bmi-1*, in protein level as well as in gene level, is favorably associated with poor prognostic markers and clinical outcome in diverse human cancers, such as colorectal cancer, nasopharyngeal carcinoma, bladder cancer and myelodysplastic syndrome [14,17-19]. However, to our best knowledge, there is little information about prognostic status and clinical outcome of *Bmi-1 *expression in ovarian cancer. This is the first study evaluating the expression of *Bmi-1 *by IHC, in association with clinicopathological and prognostic significance for a large number of ovarian cancer patients. Consistent with previous reports of other types of human cancer, in this study, we found that intensive expression of *Bmi-1 *in ovarian carcinoma was a predictor of short overall survival, independent of stage and grade. These findings raise the question of a potentially why important role of *Bmi-1 *as an underlying biological mechanism in the development and/or growth of human cancers.

It is known, the encoded protein of *Bmi-1*, as well as other proteins from the PcG family, can block the transcription of some genes such as p16Ink4a and p19Arf involved in tumour suppression, resulting in oncogenic effects [14]. In our previous investigation, however, we found that Bmi-1 may promote immortalization of nasopharyngeal carcinoma by modulating the expression of other genes, besides regulating p16Ink4a. Also, in the present study, we did not observe a significant correlation between Bmi-1 expression and either p16Ink4a or p14ARF expression in ovarian carcinoma cohorts. This data provided evidence that Bmi-1 does act through other molecular targets than repression of p16Ink4a/p14ARF in ovarian carcinogenesis. Recently, it was reported that by applying a mouse/human comparative translational genomics approach, a Bmi-1-driven 11-gene signature was identified. This cohort of 11 genes was confirmed to be a magic marker of stem cell-ness and therapy failure in patients with a variety of aggressive tumors [18]. Clearly, further work needs to be done to more precisely understand the molecular mechanism of *Bmi-1 *in the development and progression of ovarian carcinoma, as well as other human cancers.

With regard to the mechanism of up-regulated protein expression of *Bmi-1 *in ovarian carcinomas, it is known that gene amplification is a common pathological mechanism of gene overexpression in human cancers [30]. To determine whether the overexpression of *Bmi-1 *in ovarian carcinomas was caused by gene amplification, the amplification status of *Bmi-1 *was examined by FISH. In our 96 informative cases of ovarian carcinomas by both IHC and FISH simultaneously, intensive expression of *Bmi-1 *was detected in all (8/8) ovarian carcinomas that had *Bmi-1 *amplification. However, amplification of *Bmi-1 *was not observed in 28 other ovarian carcinomas with intensive expression of Bmi-1. These results indicate that the expression level of Bmi-1 protein in ovarian carcinoma does not always coincide with gene amplification. In addtion, in bladder cancers, a significant difference in Bmi-1 protein expression and in mRNA levels was obtained, but Bmi-1 protein was up-regulated to a much greater extent than Bmi-1 mRNA in cancer tissue compared with non-cancerous tissues, implying that the major source of Bmi-1 expression might be dysregulation at the post-transcriptional level in bladder cancers [19]. These data suggest that the up-regulation of protein expression of *Bmi-1 *in human cancers is complicated and it might be regulated not only by gene amplification, but also by other molecular mechanisms including transcriptional regulation and post-translational regulation.

## Conclusion

In summary, in this study, we describe, for the first time, protein expression and amplification patterns of *Bmi-1 *in normal human ovary, benign, borderline and malignant epithelial ovarian tumor tissues. Our results provide a basis for the concept that increased expression of Bmi-1 in human ovarian carcinoma may be important in the acquisition of an invasive and/or aggressive phenotype. In addition, our study introduces Bmi-1 expression as a new independent prognostic marker in ovarian carcinoma with intensive expression of Bmi-1 protein in tumor cells predicting poor outcome of the disease for the individual patient.

## Competing interests

The authors declare that they have no competing interests.

## Authors' contributions

GFY evaluated the clinical records and drafted the manuscript. WPH carried out the immunohistochemistry assays and help to draft the manuscript. MYC participated in the statistical analysis and participated in its coordination. HLR performed the immunohistochemical analyses. JHL and HXD help to carry out the immunohistochemistry assays. XYG, MSZ, YXZ and DX participated in the design of the study, in its analysis and in the interpretation of the data. DX designed the study and also participated in evaluated the immunohistochemistry results and wrote the manuscript. All authors read and approved the final manuscript.

## Pre-publication history

The pre-publication history for this paper can be accessed here:

http://www.biomedcentral.com/1471-2407/10/133/prepub
